# Recent advances in the design of single-atom electrocatalysts by defect engineering

**DOI:** 10.3389/fchem.2022.1011597

**Published:** 2022-09-15

**Authors:** Wei Li, Zhikai Chen, Xiaoli Jiang, Jinxia Jiang, Yagang Zhang

**Affiliations:** ^1^ State Key Laboratory of Electronic Thin Films and Integrated Devices, School of Materials and Energy, University of Electronic Science and Technology of China, Chengdu, China; ^2^ Chongqing Medical and Pharmaceutical College, Chongqing, China

**Keywords:** single-atom catalysts, defect engineering, defective carbon-based support, defective metal-based support, electrocatalytic reaction

## Abstract

Single-atom catalysts (SACs) with isolated metal atoms dispersed on supports have attracted increasing attention due to their maximum atomic utilization and excellent catalytic performance in various electrochemical reactions. However, SACs with a high surface-to-volume ratio are fundamentally less stable and easily agglomerate, which weakens their activity. In addition, another issue that restricts the application of SACs is the low metal loading. Defect engineering is the most effective strategy for the precise synthesis of nanomaterials to catch and immobilize single atoms through the modulation of the electronic structure and coordination environment. Herein, in this mini-review, the latest advances in designing SACs by defect engineering have been first highlighted. Then, the heteroatom doping or intrinsic defects of carbon-based support and anion vacancies or cation vacancies of metal-based supports are systematically evaluated. Subsequently, the structure–activity relationships between a single-atom coupled defect structure and electrocatalytic performance are illustrated by combining experimental results and theoretical calculations. Finally, a perspective to reveal the current challenges and opportunities for controllable preparation, *in situ* characterization, and commercial applications is further proposed.

## Introduction

With the increasing depletion of fossil fuels (e.g., coal, oil, and natural gas), exploiting renewable, sustainable, and eco-friendly fuels is quite urgent to alleviate the energy crisis in modern society (Mi et al., 2018; Guo and Zhang, 2020). At present, increasing attention is being devoted to electrochemical energy conversion and storage (e.g., fuel cells, water-splitting devices, storage batteries, artificial carbon, and nitrogen fixation) due to their high energy densities and environmental benefits (Wang et al., 2019; Ai et al., 2021; Chen et al., 2021). The operation of all of these technologies requires appropriate advanced electrocatalysts to reduce the energy barriers and accelerate the kinetics of chemical reactions, such as the hydrogen oxidation reaction (HOR), oxygen reduction reaction (ORR), hydrogen evolution reaction (HER), oxygen evolution reaction (OER), nitrogen reduction reaction (NRR), carbon dioxide reduction reaction (CO_2_RR), biomass oxidation reaction, and so on (Jiang et al., 2020; Zhou et al., 2020; Li et al., 2021a; Wang et al., 2022b; Wang et al., 2022c; Zhu et al., 2022). Accordingly, the state-of-the-art catalysts mainly depend on platinum group metals (PGMs, Pt, and Au), PGM-based oxides (IrO_2_ and RuO_2_), and PGM-based alloys (PtRu and PtPd) (Li et al., 2022; Yan et al., 2022; Zhao et al., 2022). Regrettably, the apparent activity, limited durability, high cost, and low storage of PGM-based electrocatalysts have significantly hampered the commercialization process. The intuitive strategy to overcome this bottleneck issue is to use single-atom catalysts (SACs), which not only profoundly expose the atoms to reduce the usage of PGMs but also trigger the immeasurable performance on account of the size effect and isolation effect (Qiao et al., 2011; Wang et al., 2021). However, the atomically dispersed metal atoms often inevitably suffer from migration and agglomeration on support (Xu et al., 2018). The reason for this is that SACs with a high surface-to-volume ratio are thermodynamically less stable. In addition, the high loading of SACs is also a great challenge for practical applications (Zhu et al., 2017; Sun et al., 2021). Therefore, the development of efficient and robust SACs with high atomic density and dispersion is still a challenge.

From the second law of thermodynamics, defects widely exist in nanomaterials. They could disturb or even break the periodic structure of crystals (Yan et al., 2017; Li et al., 2020). Subsequently, the electronic structure of the nanomaterials is redistributed, and then the adsorption energy of intermediate species is optimized. Consequently, the kinetics of catalytic reactions is accelerated (Yan et al., 2019). In addition to activating the intermediate species, defect engineering can be also used as an effective strategy to alter the surface-interfacial coordination environment of the supports to form strong intercalation for the capture of the metal species, such as particles, clusters, and even single atoms. Moreover, some defects (e.g., vacancies and edge sites) serve as an adsorbed site to catch and disperse single atoms. Such an excellent defective structure, by benefiting the capture of a single atom, leads to an extraordinarily high metal loading (∼20%) in SACs, which exceeds that of the traditional strategy with a very low metal loading of 1–2 wt% (Cheng et al., 2018). Furthermore, the defects and SACs synergistically improve the electrocatalytic performance in terms of selectivity, activity, and durability (Zhang et al., 2019b; Ban et al., 2021; Zhang et al., 2021). However, a deep understanding of the role of defects and SACs in the catalytic process is still lacking, and the interrelation between defects and SACs in synthetic catalysts is poorly recognized. More recently, several *in situ* spectral and microscopical instruments provide more in-depth information about the nanomaterials at the molecular and atomic levels, such as studying the growth process of electrocatalysts, observing the electrochemical reaction process directly, and analyzing the reaction mechanisms accurately (Zhang et al., 2019a; Wang et al., 2022a). These findings encourage us to further explore designing SACs by defect engineering.

Herein, in this mini-review, we first systematically summarize the latest advances in designing SACs by defect engineering. Subsequently, we turn our attention to addressing how the defective carbon-based supports (e.g., heteroatom doping and intrinsic defects) and defective metal-based supports (e.g., cation vacancies and anion vacancies) affect the formation of SACs. Then, the structure–activity relationships between a single-atom coupled defect structure and electrocatalytic ability are further discussed. At the end of this review, after an in-depth understanding of the single atom and defect structure, the prospects and challenges of defective SACs are proposed in terms of controllable preparation, *in situ* characterization, and practical application.

## Defect engineering on carbon-based materials

Owing to their low prices, excellent electrical conductivity, high specific surface area, and robust stability in both acidic and alkaline electrolytes, carbon-based materials have been selected as the most routine support (Yang et al., 2017; Li et al., 2018). However, because of the different specific surface energy of metal and carbon, the weak interactions between single metal atoms and carbon support could cause the migration and aggregation of single metal atoms, and thus the electrocatalytic performance becomes poor. To solve these issues, significant interest has been devoted to the design and synthesis of defective carbon-based materials to disperse single metal atoms due to their controllable coordination-unsaturated environment and strong metal–support interactions (Tang et al., 2020; Khan et al., 2021). Heteroatom doping and intrinsic defects are the main defective carbons, which not only regulate the surface charge and electronic structure of carbons but also offer anchor sites for the immobilization of single metal atoms. Benefiting from such a unique structure, the isolated metal atoms should be firmly captured, and the catalytic performance of catalysts could be further improved. In this section, the recent studies of single atomic electrocatalysts supported on defective carbon support have been highlighted.

**FIGURE 1:**
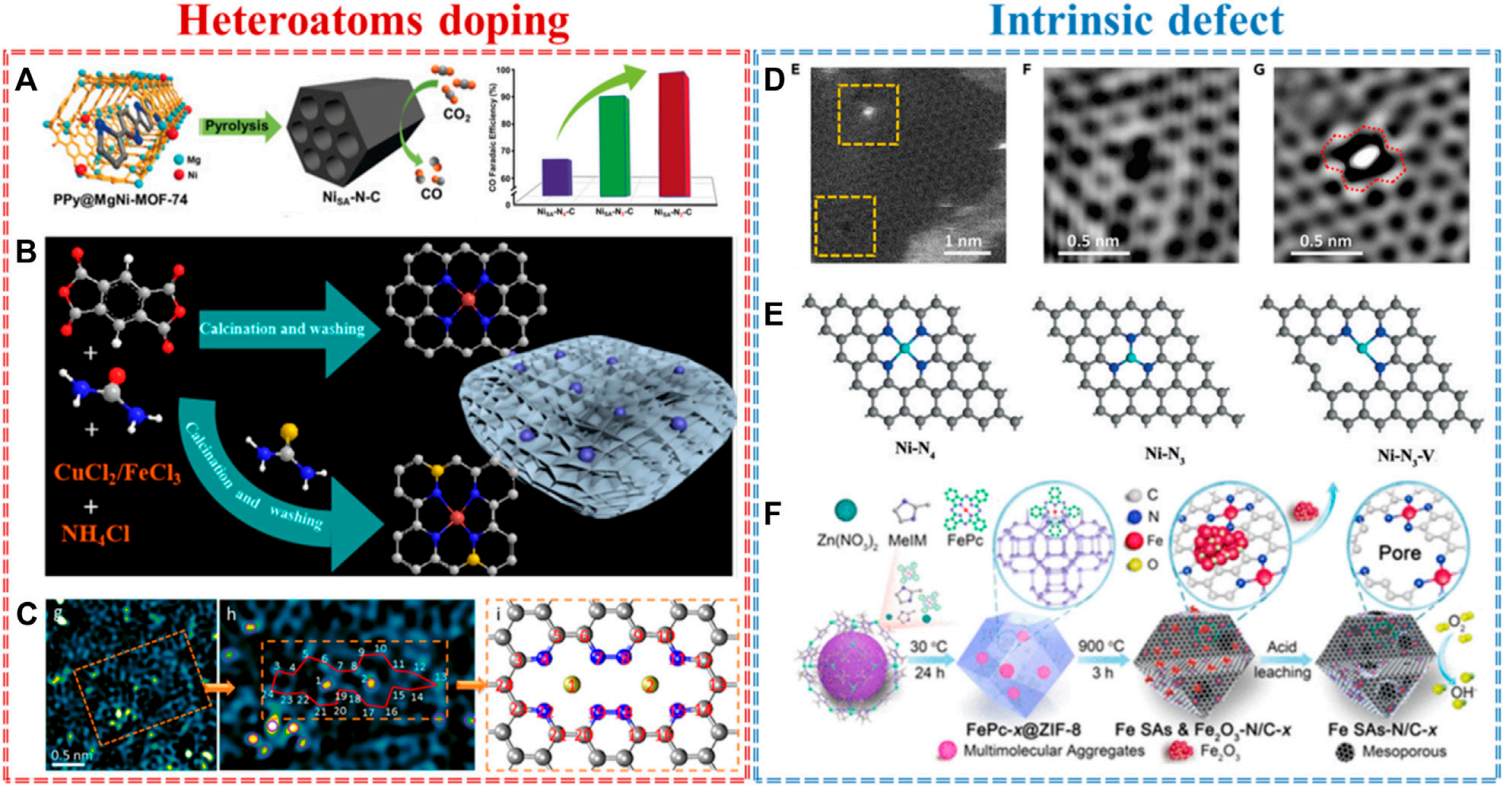
Heteroatom doping: **(A)** single atomic Ni–Nx–C (x = 2, 3, and 4) for CO_2_RR (Gong et al., 2020), **(B)** Cu/Fe–N(S)C SACs with defect-rich carbon for electrochemical denitrification (Li et al., 2021), **(C)** atomic Co-Pt supported on N doped carbon for ORR (Zhang et al., 2018a). Intrinsic defects: **(D)** graphene defects trap atomic Ni species for HER and OER (Zhang et al., 2018b), **(E)** vacancy-defect Ni SACs for CO_2_RR (Rong et al., 2020), and **(F)** edge-hosted Fe-N_4_ moieties in Fe–N–C SACs for ORR (Jiang et al., 2018).

Single atomic metal–nitrogen–carbon has been widely studied as one of the most promising alternatives to PGM-based electrocatalysts for different electrochemical applications. The species, loading, and even the adjacent coordination environment of the single metal atom play a part in their performance (Zhao et al., 2021). Heteroatom doping is the most common defect in nanocarbon materials. Based on different electronegativities and atomic sizes, heteroatom (e.g., N, C, B, P, S, and O) defects can effectively alter the coordination structure of the single metal atom. For example, Gong and co-workers developed a single-Ni-atom-implanted nitrogen-doped carbon (NC) using a host–guest cooperative protection strategy (Gong et al., 2020). X-ray absorption spectroscopy (XAS) revealed that the coordination structure of Ni–N is finely tuned by the C-doping defect from Ni–N_4_ to Ni–N_3_C_1_ and Ni–N_2_C_2_. The electrocatalytic CO_2_ reduction tests exhibited that Ni–N–C with the lowest N coordination number (Ni–N_2_C_2_) presents the highest CO Faradaic efficiency of 98% and turnover frequency of 1,622 h^−1^, far superior to those of Ni–N_3_C_1_ and Ni–N_4_ (Figure 1A). Density functional theory (DFT) calculations showed that the doping defect in Ni–N_2_C_2_ could optimize the coordination number of Ni–N and then facilitate the formation of a COOH* intermediate, consequently improving their activity and selectivity. In addition to adjusting the coordination structure of single metal atoms, heteroatom doping can also regulate the electronic structure of single metal atom-based active sites. Li and co-workers synthesized Cu or Fe single atom anchored on an S, N-codoped carbon basal plane by a defect trapping strategy (Figure 1B) (Li et al., 2021a). Both Raman spectra and electron paramagnetic resonance (EPR) spectroscopy displayed that S-doping created numerous defects in NSC compared to that in NC. Experimentally, the Fe-NSC catalyst achieves the maximum NH_4_^+^ yield of 7.822 g.N.g^−1^Fe, Faradaic efficiency of 78%, and lower charge-transfer resistance. XAS and theoretical calculations illustrated that S-doping induces an asymmetric charge distribution and redistribution, thus increasing the interaction between single atom Fe and defect carbon and resulting in efficient electrochemical denitrification. Apart from dispersing monometallic atoms, bimetallic atoms could be also anchored by doping defects. Zhang and co-workers reported a double atomic Co-Pt site supported on an N-doped defective carbon (denoted as A-CoPt-NC) by electrochemical activation (Zhang et al., 2018a). The EXAFS data and transmission electron microscopy (TEM) image showed that the N-doping defect takes part in capturing a single Co atom and then benefits from coordinating with Pt (Figure 1C). The optimal catalysts exhibited ultrahigh ORR performance and 267 times higher mass activity compared to commercial Pt/C. Theoretically, this dramatic improvement in ORR activity is ascribed to the synergetic effects of bimetallic atom (Co-Pt) and defect supports, which alters the d-orbital shift and the coordination structures of atomic metals.

After a deeper understanding of doping defects, we found that heteroatoms play an important role in activating adjacent carbon atoms to improve the performance of electrocatalysts, revealing that the truly active sites are carbon atoms themselves. Thus, we have reasons to believe that the intrinsic defects in carbon-based supports are more precious than heteroatom doping. For example, Zhang and co-workers constructed atomically dispersed Ni atoms trapped on graphene defects (A-Ni@DG) by a facile acid-leaching strategy (Zhang et al., 2018b). Both the HAADF-STEM image (Figure 1D) and XAS results showed that the intrinsic defect in graphene not only serves as an anchored site to capture a single Ni atom but also tailors the electronic densities of state (DOSs) of Ni species. Such an excellent structure, by precisely controlling the coordination environment of Ni, leads to exceptionally high performance in electrochemical water-splitting with an ultralow overpotential of 70 mV for HER and 270 mV for OER at 10 mA cm^−2^, which surpasses that of the state-of-the-art Pt/C and Ir/C catalysts. As well as pure intrinsic defects, vacancy defects are widely studied in SACs. For example, Chen and co-workers prepared a vacancy-defect Ni–N_3_–V single-atom catalyst by cleaving the weaker Ni–O interaction in the precursors of Ni–N_3_O–C at a high temperature (Rong et al., 2020). The Ni–N_3_–V showed an extremely high Faradaic efficiency (over 90%) and a record high turnover frequency (1.35 * 10^5^ h^−1^), which is much higher than those of Ni–N_4_ SACs. This enhancement is owed to the vacancy defect in Ni–N–C, which increases the CO* desorption energy of active sites (Figure 1E). Not only vacancy defects but also the edge sites in SACs could affect the coordinate structure of metal-N_4_. Jiang and co-workers built an atomically dispersed Fe-N_4_ site embedded in the edge of three-dimensional (3D) hierarchically porous carbon by encapsulating iron (II) phthalocyanine (FePc) into the cavity of ZIF-8 by edge-site engineering (Jiang et al., 2018). The micro–mesoporous carbon is formed by the aggregation of excessive FePc molecules through the Kirkendall effect, and subsequently, the Fe–N_4_ site is *in situ* generated on the edge of pore carbon (Figure 1F). The synergistic effect between single metal sites and defective carbons delivers superior ORR performance with a half-wave potential of 0.915 V and a high atom-utilization efficiency up to a 10-fold enhancement compared to any Fe-based catalyst. DFT calculations further demonstrated that edge-N atoms play a vital role in tuning the surface charge of the Fe–N_4_ structure and then optimizing the adsorption energy of oxygen species to improve the ORR activity. In general, these results demonstrate that heteroatom doping and intrinsic defects benefit the formation of strong interfacial interactions between single metal atoms and the defective carbon, finely controlling the electronic structures and the coordination environment of the single-atom catalysts for electrocatalysis.

## Defect engineering on metal-based materials

As summarized earlier, carbon-based defective materials are cheap and a fine support for capturing single atoms. However, the corrosion of carbon under high potentials is an inevitable problem in the electrocatalytic process, which probably induces the collapse of catalysts (Xie et al., 2014; Qiao et al., 2019). Recently, due to facile syntheses and stability in high potentials, increasing attention has been paid to metal-based materials, such as metal oxide, metal nitride, metal sulfide, and metal phosphide. Meanwhile, highly dispersed SACs have been well anchored on metal-based supports by various defects, such as cation vacancies and anion vacancies (Tan et al., 2022; Xiao et al., 2022). In this part, we turn our focus to metal-based defective materials stabilizing single metal atoms.

**FIGURE 2:**
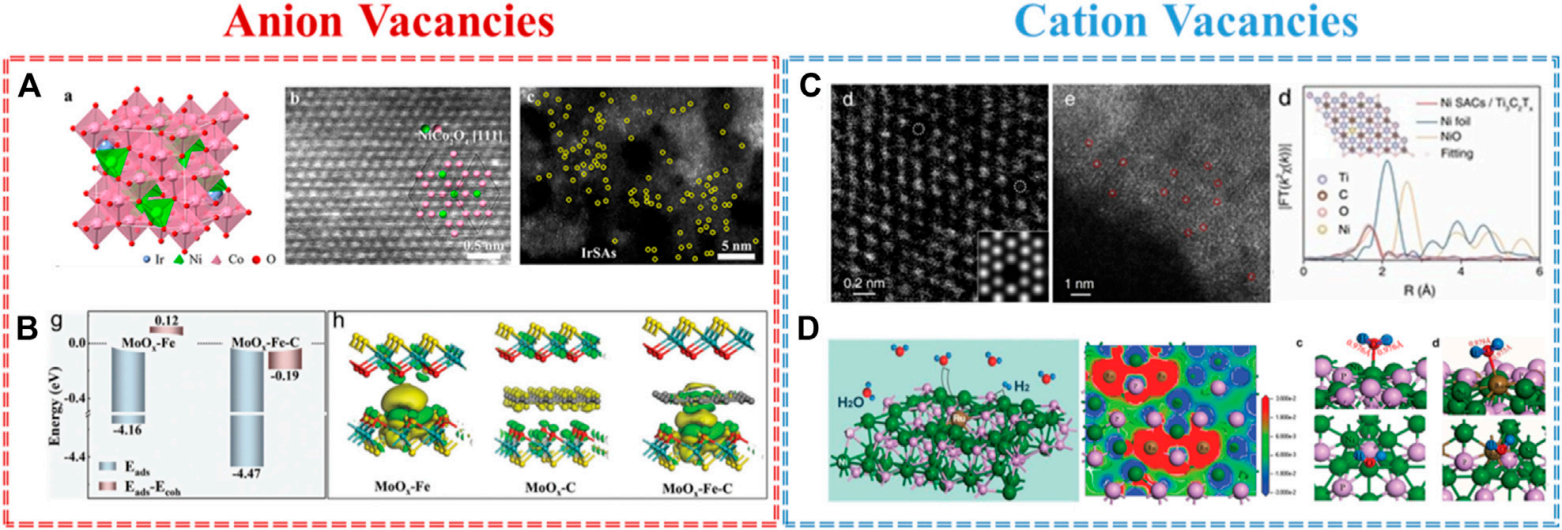
Anion vacancies: **(A)** Ir SACs supported on O vacancy in NiCo_2_O_4_ for OER (Yin et al., 2020) and **(B)** Fe SACs supported on S vacancy in MoS_2_-C for HER and OER (Gong et al., 2022). Cation vacancies: **(C)** Ni SACs supported on Ti vacancy in MXene for hydrazine oxidation reaction (Zhou et al., 2022) and **(D)** Ru SACs supported on Ni vacancy in Ni_5_P_4_ for HER (He et al., 2020).

The most common and popular anion vacancies in metal-based materials are oxygen vacancies. Benefiting from the low formation energy of oxygen vacancies, the physicochemical properties of defective metal oxides may be changed, and the oxygen vacancies could be also used as the anchored sites to capture single atoms. For example, Yin and co-workers demonstrated a co-electrodeposition method to prepare Ir single atom coupling with oxygen vacancies on ultrathin NiCo_2_O_4_ porous nanosheets (Ir-NiCo_2_O_4_ NSs) (Yin et al., 2020). Figure 2A further verifies the defect structure and single atom of Ir-NiCo_2_O_4_ NSs by HAADF-STEM. The lower coordinated Co sites near the oxygen vacancies play an important role in anchoring atomic Ir and then facilitate electron exchange and transfer. In addition, the Ir-Ox sites not only protect the chemical state of Co during the OER but also optimize the adsorption energy of H and O on the Co site, which could ensure the utilization of Co-based activity sites. As a result, Ir-NiCo_2_O_4_ NSs showed an excellent OER activity of 240 mV overpotential at 10 mA cm^−2^ and robust stability of 70 h under the condition of acid electrolyte. Additionally, Tong and co-workers documented that the defect-rich W_18_O_49_ support could easily catch the single Fe atom by generating oxygen vacancy for NRR (Tong et al., 2020). Zhang and co-workers fabricated oxygen vacancy MXene to stabilize atomic Pt for HER (Zhang et al., 2022). Except for oxygen vacancy, the sulfur vacancy-based materials also act well for capturing single atoms. Gong and co-workers reported the simultaneous modulation of the mesoscale diffusion and Mo-Fe-C active site formation over monodispersed hollow Fe@MoS_2_-C sub–microreactors (Gong et al., 2022). A unique microenvironment, used by the sulfur vacancies and intercalated carbon, could catch Fe single atom to form the Fe@MoS_2_-C sub–microreactor. Owing to the rich sulfur vacancies and mesoscale diffusion, the Fe@MoS_2_-C exhibited an improved OER performance compared to the Fe-based SACs reported in the data. The theoretical calculation revealed the stability of Mo–Fe–C coordination, the electron transfer channel of “MoOx → Fe → carbon,” and the favorable d-band center in the dual-anchoring model (Figure 2B).

In addition to the anion vacancies, the cation vacancies are also delivered to engineer the high-performance SACs because of their various electron and orbital distributions. For instance, Zhou and co-workers prepared titanium vacancy as structural defects that serve to catch and anchor Ni single atoms by a ‘self-reduction’ strategy (Zhou et al., 2022). The cation vacancy-rich Ti_3_C_2_Tx MXene acts simultaneously as a support and reductant during the nucleation of Ni SACs. Figure 2C further confirms the structure of Ni NPs/Ti3C2Tx in terms of the cation vacancies in Ti3C2Tx, the Ni single atom trapped on Ti vacancies, and the coordinate structure of Ni in Ni NPs/Ti3C2Tx. Experimentally, the Ni NPs/Ti3C2Tx catalysts exhibited an ultralow onset potential of -0.03 V (vs. RHE) and a negligible activity loss during a stability test of 24,000 s. Theoretically, this dramatic increase in hydrazine oxidation reaction activity is ascribed to the redistribution of electronic density of states by the strong interaction between the Ni single atom and adjacent C atoms. Subsequently, He and co-workers constructed nickel-vacancy-rich nickel hydroxides as a support precursor to capture the atomic Ru and for the subsequent phosphorization treatment to obtain Ni_5_P_4_-Ru (He et al., 2020). The physical characterizations, such as XAS, STEM, and EPR results, affirmed the strong coupling between Ni vacancies and Ru single atoms. Benefiting from the single atom coupled vacancies defect, Ni_5_P_4_-Ru achieves an ultralow overpotential of 54 mV at 10 mA cm^−2^ and long-term stability for OER. Both meticulous spectroscopic analysis and theoretical calculations revealed that single Ru atom coupled Ni vacancies could induce localized structure polarization, which decreases the reaction barrier of water dissociation and optimizes the hydrogen adsorption free energy on Ru sites (Figure 2D). Generally speaking, understanding the positive effect of cation vacancies and anion vacancies in metal-based materials provides further direct evidence and information to guide us toward a rational and precise SAC design at the molecular and atomic levels.

## Conclusion and outlook

SACs, as economical alternatives to noble metal-based catalysts, presented excellent catalytic performances for various electrochemical applications because of their well-defined active centers, maximum atomic utilization, explicit coordinate structure, and strong single-atom–support interactions. However, SACs with a high surface-to-volume ratio are thermodynamically less stable. Herein, in this mini-review, we paid attention to the high-efficiency capture and immobilization of SACs by defect engineering. Defects not only tailor the surrounding electronic structure and coordination environment of support but also serve as the anchor sites to catch the isolated single atom and prevent the migration and agglomeration of SACs. Consequently, the electrocatalytic performance is further enhanced by defect engineering and single-atom catalysts simultaneously. Additionally, with the development of synthesis strategies, characterization techniques, and theoretical modelings, the defective SACs have achieved significant progress in recent decades. However, there are always many difficulties and challenges to be explored.

### (i) Precisely controlling the formation of SACs by defect engineering

The electrocatalytic performances of SACs are limited by the loading of single atoms. However, the conventional strategies for the construction of SACs are usually with low metal loading due to their extremely high surface energy. Defect engineering could tune the electronic structure of support and then optimize the adsorption energy of anchored sites to facilitate the capture of single metal atoms. Based on the strong interaction between defects and single atoms, an increasing number of isolated metal atoms can be trapped and stabilized on the defective support to achieve a high metal loading. Therefore, the exploration of defective supports benefits carrying out the high metal loading in SACs and provides a new opportunity for maximum utilization of catalysts.

### (ii) Exploring the structural evolution of defective SACs via advanced characterization methods under the operational process

Harsh electrocatalytic processes (e.g., long-term operation in high potentials, strongly acidic or alkaline electrolyte) may induce the structural evolution of defective SACs. In addition, the structural evolutions of active sites in previous research studies are almost from theoretical simulations. Thus, it is quite urgent to develop advanced characterization techniques to analyze and study the evolution of active sites in the electrocatalytic reaction directly. Coupling with experimental results and theoretical calculation will provide more invaluable information for further detecting the truly active site under the operational process.

### (iii) Large-scale synthesis of defective SACs for practical application

In the traditional fabrication of SACs, the routine precursors are metal–organic frameworks, and the conventional approach is atomic layer deposition. In addition, the generation of defects in supports is a high-energy process. Thus, the synthesis cost, yields, and efficiency of defective SACs are the major challenges, which limit the actual production. Accordingly, the development of an efficient strategy for large-scale synthesis of defective SACs is vital to satisfy the potential demands of their practical application.
